# Potential Role of Circulating miRNAs for Breast Cancer Management in the Neoadjuvant Setting: A Road to Pave

**DOI:** 10.3390/cancers15051410

**Published:** 2023-02-23

**Authors:** Chiara Benvenuti, Paola Tiberio, Mariangela Gaudio, Flavia Jacobs, Giuseppe Saltalamacchia, Sebastiano Pindilli, Alberto Zambelli, Armando Santoro, Rita De Sanctis

**Affiliations:** 1 Medical Oncology and Hematology Unit, IRCCS Humanitas Research Hospital, 20089 Rozzano, MI, Italy; 2 Department of Biomedical Sciences, Humanitas University, 20072 Pieve Emanuele, MI, Italy

**Keywords:** microRNAs, circulating miRNAs, breast cancer, neoadjuvant chemotherapy, pathological complete response

## Abstract

**Simple Summary:**

In breast cancer management, neoadjuvant chemotherapy is well established as therapeutic choice for selected high-risk early or locally advanced breast cancer. However, besides there being few clinical genomic classifiers, there is no technology that can predict for certain whether breast cancer patients will benefit from neoadjuvant chemotherapy in terms of pathological complete response and disease-free survival. The analysis of miRNAs from biological fluids at the beginning of therapy is simple and may aid in the identification of patients who will receive the greatest benefit. On the other hand, monitoring circulating miRNA levels during treatment could allow the early identification of patients who will not benefit from it (avoiding unnecessary treatments and related side effects). Therefore, there is an urgent clinical need for non-invasive biomarkers in the neoadjuvant setting, and circulating miRNAs could theoretically meet this need, but there is still a long way to go until their use in clinical practice can be established.

**Abstract:**

Recently, circulating microRNAs (miRNAs) have emerged as potential non-invasive biomarkers for breast cancer (BC) management. In the context of BC patients undergoing neoadjuvant chemotherapy (NAC), the possibility of obtaining repeated, non-invasive biological samples from patients before, during, and after treatment is incredibly convenient and provides the opportunity to investigate circulating miRNAs as diagnostic, predictive, and prognostic tools. The present review aims to summarize major findings in this setting, thus highlighting their potential applicability in daily clinical practice and their possible limitations. In all the contexts (diagnostic, predictive, and prognostic), circulating miR-21-5p and miR-34a-5p have emerged as the most promising non-invasive biomarkers for BC patients undergoing NAC. Specifically, their high baseline level could discriminate between BC patients and healthy controls. On the other hand, in predictive and prognostic investigations, low circulating miR-21-5p and miR-34a-5p levels may identify patients with better outcomes, in terms of both treatment response and invasive disease-free survival. However, the findings in this field have been very heterogeneous. Indeed, pre-analytical and analytical variables, as well as factors related to patients, may explain the inconsistency among different study results. Thus, further clinical trials, with more precise patient inclusion criteria and more standardized methodological approaches, are definitely needed to better define the potential role of these promising non-invasive biomarkers.

## 1. Introduction

Breast cancer (BC) is the most prevalent neoplasm and the leading cause of cancer-related mortality among women globally, although improvements in early diagnostic procedures and advances in treatments have contributed to decreasing the mortality rate over time [1,2,3]. Pathological variables such as tumor grade (G), TNM stage, proliferation index (Ki67), hormone receptor (HR) status, human epidermal growth factor receptor 2 (HER2) expression, and molecular subtype are well-known features that strongly influence patients’ prognoses. Together with clinical (e.g., menopausal status, age, and performance status) and biological (e.g., genomic signature) parameters, these pathological features are essential for the identification of the best-tailored treatment. 

In BC management, neoadjuvant chemotherapy (NAC) is a well-established therapeutic choice for selected high-risk early and locally advanced BC [4]. However, before starting treatment, the individual response to NAC and the subsequent long-term prognosis has been almost unpredictable to date. Likewise, an early identification of patients with poor tumor response could be crucial for avoiding unnecessary treatments and related side effects. For this reason, there is an urgent clinical need for non-invasive prognostic/predictive biomarkers in the neoadjuvant setting. A biomarker is considered prognostic if it provides information about outcomes regardless of treatment, reflecting the underlying intrinsic behavior of the disease, whereas a biomarker is considered predictive if treatment outcomes are different for biomarker-positive patients compared to biomarker-negative patients [5]. 

MicroRNAs (miRNAs) are highly conserved small noncoding RNAs formed by approximately 22 nucleotides which play key roles in gene regulatory networks. MiRNA biogenesis is a complex process involving several enzymes that first generate a primary miRNA, then a precursor miRNA (pre-miRNA) and finally a miRNA duplex composed of two strands: the 5p and the 3p (generating from the 5′ end and 3′ end of the pre-miRNA, respectively) [6]. One of the strands (the guide strand) is then loaded into the Argonaute (AGO) family of proteins, generating the miRNA-induced silencing complex (miRISC) that is able to bind transcripts, thus leading to mRNA degradation, translational suppression, or, as has been more recently discovered, an increase in the translation of target mRNA [6,7]. The other strand (the passenger strand) was initially thought to be degraded; however, it has been demonstrated that both strands can be incorporated into the miRISC complex depending on tissue or cell type [8]. The miRBase database (a searchable database of published miRNA sequences and annotations) provides information on stem-loop and mature miRNA sequences, including which is the guide strand (for which the miRBase alias is the miRNA name without any specification: miR) and which is the passenger ones (also named star strand; miRBase alias: miR*). Thus, the lack of specific information about the miRNA strand under investigation in a research paper means that the authors refer to the miRBase alias used for the guide strand. However, for a few miRNAs (e.g., miR-718, -4516, -422a, -484), it has not yet been defined in the miRBase which is the guide strand.

The discovery that miRNAs are differently expressed in normal tissue and several tumor types, including BC [9], has generated a great deal of interest in the scientific community. To date, miRNA signatures from normal tissues, cancer tissues, and metastases have been used to classify different types of cancer and have been shown to represent potential biomarkers for diagnosis, prognosis, and treatment response [10,11]. In addition, different studies have shown that miRNAs can be released from cells and enter circulation [9,10,11,12,13,14]. In fact, it has been demonstrated that miRNAs can be found in circulation as a passive consequence of apoptotic and necrotic cell death, or due to active secretion through regulated cellular processes [6,12,13,14,15,16,17]. As a result, these molecules have also been found in several human bodily fluids (including blood, serum, plasma, urine, saliva, seminal fluid, and pleural effusion) [18], in a stable form protected from endogenous RNAses (i.e., in vesicles such as exosomes, microvesicles, and apoptotic bodies, or associated with proteins such as AGO2), thus making circulating miRNA levels well suited for non-invasive analysis in patient samples [19,20,21,22,23]. Indeed, independent studies have reported the feasibility of using circulating miRNAs as potential disease biomarkers of tumor clinical-pathological variables and patient clinical outcomes, including for BC [24,25,26].

In the context of NAC for early BC, the availability of non-invasive biological samples before, during, and after treatment is strongly convenient and provides the opportunity to investigate circulating miRNAs as prognostic and predictive biomarkers for daily clinical practice. This review aims to summarize the major findings regarding circulating miRNAs as diagnostic, predictive, and prognostic biomarkers in BC patients undergoing NAC, by highlighting their potential applicability in clinical practice and their possible limitations.

## 2. Diagnostic Potential of Circulating miRNAs

In recent decades, several studies have thoroughly investigated circulating miRNAs as candidates for discriminating between BC patients and healthy controls, and possibly contributing to BC diagnosis in the case of incertitude. As reported in Table 1, the results of these case-control studies have been very heterogeneous and somewhat conflicting. 

The miRNAs most frequently cited in the literature for their potential diagnostic value are miR-21-5p and miR-155-5p (oncogene-like), and miR-let-7a-5p and miR-34a-5p (tumor suppressor-like). Strong and consistent data from multiple studies have shown that circulating miR-21-5p levels were significantly higher in the serum and plasma of BC patients compared to healthy women, regardless of subtypes [27,28,29,30,31,32]. In addition, Rodriguez-Martinez et al. reported significantly higher miR-21-5p levels in advanced BC patients compared to early BC, suggesting that circulating miR-21-5p may be a promising candidate not only for early tumor detection but also as a marker of tumor burden [32]. Similarly, the analyses of circulating miR-155-5p levels showed highly consistent results across studies, indicating a marked increase in serum miRNA levels in BC patients compared to healthy controls [30,33,34]. In contrast, for the tumor suppressor-like miRNAs miR-let-7a-5p and miR-34a-5p inconsistent results have been reported among different studies. In fact, Marques et al. reported markedly lower levels of miR-let-7a-5p in plasma and serum samples from BC patients compared to healthy controls [35]. However, in other studies significantly higher levels of miR-let-7a-5p were found in blood or serum of BC patients compared to healthy individuals [27,36]. Similarly, circulating miR-34a-5p levels were significantly higher in BC women compared to healthy volunteers in several studies [34,37,38] but, in the case-control series reported by Freres et al., the authors found opposing results [39].

**Figure 1:**
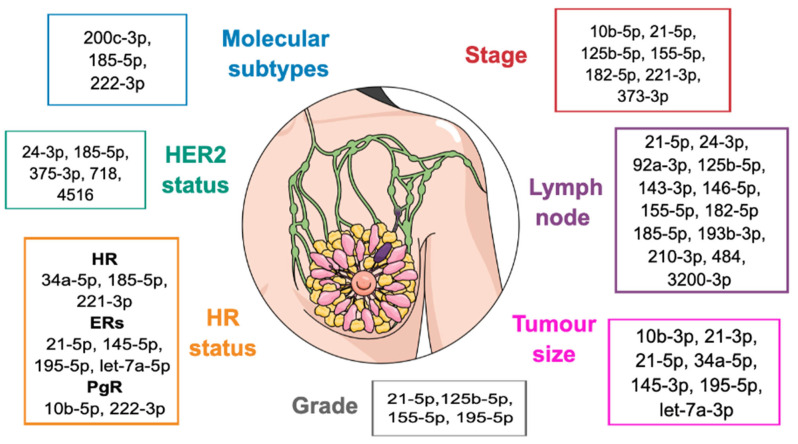
Circulating miRNAs significantly associated with BC clinical-pathological features.

As part of diagnostic evaluation, many studies then analyzed the possible correlation among circulating miRNA levels and some of the key BC clinical-pathological features (Figure 1). The strongest evidence concerns tumor grade, tumor size (T), lymph node involvement, clinical stage, molecular subtypes, HR, and HER2 expression.

Several miRNAs have been associated with tumor grade in different studies. In particular, serum miR-125b-5p levels were significantly increased in high pathologic grade compared to low-grade tumors in a series of Luminal B patients [40]. Similarly, serum miR-155-5p, plasma miR-21-5p, and blood miR-195-5p levels were higher in G3 than in G2 tumors [33,41]. 

Serum levels of miR-21-3p, miR-10b-3p, miR-145-3p, and miR-let-7a-3p were shown to correlate directly with T stage: higher levels were associated with higher tumor size [42]. In addition, serum miR-21-5p [32] and miR-34a-5p [34] levels were significantly higher in T3-T4 stages compared to T1-T2 in BC patients. Similar results were observed by Heneghan et al., who reported a significant increase in the circulating levels of miR-195-5p in blood samples of BC patients with T3-T4 tumors compared to T1-T2 ones [36]. Moreover, Stevic et al. reported that the plasma levels of six miRNAs (-185-5p, -376a-3p, -382-5p, -410-3p, -433-3p, and -628-5p) were significantly associated with a higher tumor stage (i.e., T3-T4 versus T1-T2) in a cohort of HER2-positive BC patients [43]. 

Across different studies, several circulating miRNAs have been investigated in early BC patients in association with nodal involvement. Increased levels of circulating miR-210-3p [29] in plasma and miR-125b-5p [30] and miR-21-5p [31] in serum directly correlated with the positivity of locoregional nodes. However, several other miRNAs in plasma (-24-3p, -92a-3p, -143-3p, -146-5p, -185-5p, -193b-3p, and -484) [44] and serum (-155-5p [30,45], -182-5p [45], and -3200-3p [46]) have been found to be inversely related to nodal status, with lower levels found in patients with nodal involvement. 

Multiple circulating miRNAs were also assessed for their possible relationship with clinical stage. Among them, miR-21-5p [31,45,47], miR-155-5p [30,33,45,47], miR-182-5p [45], miR-373-3p [28,48], miR-221-3p [47], miR-125b-5p [30,33,48], and miR-10b-5p [30] had significantly higher levels in both the serum and plasma of BC patients in advanced clinical stages compared to earlier stages (i.e., II versus III, or I-II versus III-IV).

Several circulating miRNAs were found to have different levels depending on the BC molecular subtype. Serum circulating miR-200c-3p levels were lower in triple-negative breast cancer (TNBC) patients compared to estrogen receptor (ER)- and progesterone receptor (PgR)-positive BC patients [49]. Conversely, Luminal-like tumors showed lower plasma miR-185-5p levels compared to TNBC [44]. Finally, Rodríguez-Martínez et al. reported definite level profiles of miRNA-222-3p in serum samples according to molecular subtype: it was decreased in Luminal A compared to basal-like and Luminal B tumors [32].

A large number of circulating miRNAs were investigated in relation to ER status. Indeed, high levels of circulating miR-221-3p in serum [50] and miR-185-5p [44] and miR-34a-5p [37] in plasma were associated with the negative expression of both ER and PgR.

Correlation among circulating miRNAs and ER was also observed for miR-195-5p (in blood samples) [41], miR-let-7a-5p [47], and miR-145-5p [48] (in plasma samples), with higher levels directly related to higher ER expression. In addition to the aforementioned results, circulating miR-221-3p, miR-185-5p, and miR-34a-5p, and serum miR-21-5p were also found to be inversely correlated with ER positivity (i.e., low circulating levels were related to higher ER expression) in a study conducted by Al-Khanbashi M and colleagues [46]. Concerning PgR expression, serum miR-222-3p [32] and miR-10b-5p [48] showed an inverse association with PgR positivity, with higher levels mostly found in PgR-negative BC.

Finally, regarding HER2 status, Zhang et al. identified three circulating miRNAs that were significantly associated with HER2 expression: serum levels of miR-375-3p, miR-718, and miR-4516 were lower in patients with HER2-negative tumors than in those with HER2-positive BC [40]. Moreover, higher plasmatic levels of miR-24-3p and miR-185-5p were associated with HER2-positive tumors [44]. Currently, there are no data on the correlation between circulating miRNA levels and low HER2 status (i.e., tumors expressing HER2 protein at the 1+ or 2+ immunohistochemistry level without HER2 gene amplification).

**Table 1:** Circulating miRNAs with a potential diagnostic role in differentiating between BC patients and healthy controls, BC molecular subtypes, and disease stage.

miRNA	Sample	BC Patients/ Healthy Controls	BC Subtype	Method	Findings	Ref
10b-3p	Plasma	30/20	All	qRT-PCR	higher levels in BC vs. HC	[42]
10b-5p	Serum	56/10	All	qRT-PCR	higher levels in BC vs. HC	[33]
	Serum	89/29	All	qRT-PCR	higher levels in advanced BC vs. HC	[34]
15a-5p	Serum	8/20	TNBC	qRT-PCR	lower levels in TNBC vs. HC	[27]
17-5p	Serum	8/20	TNBC	qRT-PCR	lower levels in TNBC vs. HC	[27]
18a-5p	Serum	8/20	TNBC	qRT-PCR	lower levels in TNBC vs. HC	[27]
19a-3p	Serum	118/30	HER2−	qRT-PCR	higher levels in BC vs. HC	[30]
19b-3p	Serum	16/20	All	qRT-PCR	lower levels in TNBC vs. HC	[27]
21-3p	Plasma	30/20	All	qRT-PCR	higher levels in BC vs. HC	[42]
21-5p	Serum	53/8	Not specified	qRT-PCR	higher levels in BC vs. HC higher levels in advanced BC vs. early BC	[32]
	Serum	8/20	All	qRT-PCR	higher levels in TNBC vs. HC	[27]
	Serum	127/19	HER2+	qRT-PCR	higher levels in BC vs. HC	[28]
	Plasma	29/28	HER2+	qRT-PCR	higher levels in BC vs. HC	[29]
	Serum	118/30	HER2−	qRT-PCR	higher levels in BC vs. HC	[30]
	Serum	75/75	Not specified	qRT-PCR	higher levels in BC vs. HC	[31]
27a-3p	Plasma	435/20	HER2+ and TNBC	qRT-PCR	higher levels in BC vs. HC lower levels in TBNC vs. HER2	[43]
27b-3p	Plasma	435/20	HER2+ and TNBC	qRT-PCR	higher levels in BC vs. HC lower levels in TBNC vs. HER2	[43]
29a-3p	Plasma	29/28	HER2+	qRT-PCR	higher levels in BC vs. HC	[29]
29c-3p	Serum	76/52	All	qRT-PCR	higher levels in BC vs. HC	[51]
30b-5p	Serum	16/20	All	qRT-PCR	lower levels in TNBC vs. HC	[27]
34a-5p	Serum	39/10	All	qRT-PCR	higher levels in BC vs. HC	[37]
	Plasma	59/20	All	qRT-PCR	lower levels in BC vs. HC	[39]
	Serum	86/20	HER2−	qRT-PCR	higher levels in BC vs. HC	[38]
	Serum	89/29	All	qRT-PCR	higher levels in advanced BC vs. HC	[34]
105-5p	Serum	53/8	Not specified	qRT-PCR	higher levels in BC vs. HC	[32]
122-5p	Plasma	59/20	All	qRT-PCR	higher levels in BC vs. HC	[39]
125b-5p	Serum	118/30	HER2−	qRT-PCR	higher levels in BC vs. HC	[30]
126-3p	Plasma	29/28	HER2+	qRT-PCR	higher levels in BC vs. HC	[29]
145-3p	Plasma	30/20	All	qRT-PCR	lower levels in BC vs. HC	[42]
155-5p	Serum	56/10	All	qRT-PCR	higher levels in BC vs. HC	[33]
	Serum	118/30	HER2−	qRT-PCR	higher levels in BC vs. HC	[30]
	Serum	89/20	All	qRT-PCR	higher levels in BC vs. HC	[34]
181a-3p	Plasma	30/20	All	qRT-PCR	higher levels in BC vs. HC	[42]
195-5p	Serum	210/102	All	qRT-PCR	lower levels in BC vs. HC	[52]
	Blood	83/63	Not specified	qRT-PCR	higher levels in BC vs. HC	[36]
	Serum	72/72	All	qRT-PCR	lower levels in BC vs. HC	[35]
199a-5p	Serum	76/52	All	qRT-PCR	higher levels in BC vs. HC	[51]
205-5p	Serum	118/30	HER2−	qRT-PCR	higher levels in BC vs. HC	[30]
210-3p	Serum	127/19	HER2+	qRT-PCR	higher levels in BC vs. HC	[28]
	Plasma	29/28	HER2+	qRT-PCR	higher levels in BC vs. HC	[29]
221-3p	Plasma	93/32	All	qRT-PCR	higher levels in BC vs. HC	[50]
335-5p	Plasma	435/20	HER2+ and TNBC	qRT-PCR	higher levels in TNBC vs. HC higher levels in TNBC vs. HER2+	[43]
365a-3p	Plasma	435/20	HER2+ and TNBC	qRT-PCR	higher levels in HER2+ BC vs. HC lower levels in TNBC vs. HER2+	[43]
376c-3p	Plasma	435/20	HER2+ and TNBC	qRT-PCR	higher levels in TNBC vs. HC higher levels in TNBC vs. HER2+	[43]
373-3p	Serum	127/19	HER2+	qRT-PCR	higher levels in BC vs. HC	[28]
	Serum	118/30	HER2-	qRT-PCR	higher levels in BC vs. HC	[30]
382-5p	Plasma	435/20	HER2+ and TNBC	qRT-PCR	higher levels in TNBC vs. HC higher levels in TNBC vs. HER2+	[43]
422a	Plasma	435/20	HER2+ and TNBC	qRT-PCR	lower levels in HER2+ BC vs. HC higher levels in TNBC vs. HER2+	[43]
424-5p	Serum	76/52	All	qRT-PCR	higher levels in BC vs. HC	[51]
433-3p	Plasma	435/20	HER2+ and TNBC	qRT-PCR	higher levels in TNBC vs. HC higher levels in TNBC vs. HER2+	[43]
451-5p	Serum	118/30	HER2−	qRT-PCR	lower levels in BC vs. HC	[30]
628-5p	Plasma	435/20	HER2+ and TNBC	qRT-PCR	lower levels in HER2+ vs. HC higher levels in TBNC vs. HER2+	[41]
let-7a-5p	Serum	8/20	TNBC	qRT-PCR	higher levels in TNBC vs. HC	[27]
	Serum	72/72	All	qRT-PCR	lower levels in BC vs. HC	[35]
	Blood	83/63	Not specified	qRT-PCR	higher levels in BC vs. HC	[36]
let-7a-3p	Plasma	30/20	All	qRT-PCR	lower levels in BC vs. HC	[42]
let-7e-5p	Serum	8/20	TNBC	qRT-PCR	higher levels in TNBC vs. HC	[27]

Abbreviations: BC: breast cancer; HC: healthy control; HER2: human epidermial growth factor 2; TNBC: triple-negative breast cancer; qRT-PCR: quantitative real-time polymerase chain reaction; Ref: reference.

## 3. Predictive Potential of Circulating miRNAs

For the prediction of clinical and pathological outcomes in an NAC setting, a plethora of circulating miRNAs have been investigated (Table 2). Here, we report the most extensively researched ones, underlining their potential clinical relevance. 

Among them, miR-21-5p has emerged as an independent predictor of response in several studies. In fact, low levels of circulating miR-21-5p before NAC (based on paclitaxel and doxorubicin) have been associated with a higher likelihood of favorable response to NAC in HR-positive BC patients [44]. Similarly, in a multicentric prospective study by McGuire and colleagues, which assessed a predefined panel of circulating miRNAs (selected on their reported relevance in BC) as a measure of NAC response in all BC subtypes, the whole-blood miR-21-5p levels of responders (defined as patients who had a complete response or >90% reduction in primary T) were considerably lower than those of non-responders (defined as patients with <90% reduction in primary T) in the HR-positive subtype (*p* = 0.048). On the contrary, in TNBC and HER2-positive BC, the response to NAC was not associated with circulating levels of miR-21-5p [41]. Likewise, similar results were reported in a Ukrainian retrospective study, which included 182 patients with stage II–III HR-positive/HER2-negative BC undergoing NAC with polychemotherapy with fluorouracil, doxorubicin, and cyclophosphamide or doxorubicin/cyclophosphamide. Patients with chemo-sensitive tumors (evaluation of NAC response performed every two cycles by mammography) showed changing in serum baseline levels of miR-21-5p lower than two-fold, while those with resistant tumors had a change above three-fold [45]. In the same study, serum miR-205-5p levels increased more than four-fold in chemotherapy-sensitive patients with the HR-positive/HER2-negative subtype and decreased lower than 2.5-fold in patients with a poorer response [45]. In contrast, another observational study with a similar cohort of patients (68 luminal A stage II-III BC patients for the discovery group, and 56 patients for the validation one) revealed that serum miR-205-5p levels in patients undergoing epirubicin- and paclitaxel-based NAC were higher in the resistant group (defined as no or minor reduction (≤30%)) compared to the sensitive group (defined as a decrease of >30% or no residual invasive cancer; *p* < 0.05) [53]. 

Besides miR-21-5p and miR-205-5p, miR-375-3p has also been shown to be a promising predictive biomarker whose basal levels are associated with response. Zhang et al. prospectively investigated the role of several miRNAs in 37 luminal B BC patients undergoing NAC with taxane- and/or anthracycline-based regimens, plus trastuzumab for HER2-positive cases. In luminal B HER2-negative patients, relatively low baseline serum levels of miR-375-3p were found to be associated with pathological complete response (pCR) (*p* = 0.043) and “comprehensive response”, defined as partial or complete response in clinical evaluation and loss > 30% or no residual invasive cancer in pathological evaluation (*p* = 0.023) [40]. Similar results were observed in the luminal A subtype in the abovementioned Ukrainian study, where decreased baseline levels predicted sensitivity to NAC with fluorouracil, doxorubicin, and cyclophosphamide, or doxorubicin and cyclophosphamide [45]. On the contrary, in the study by Wu and colleagues, when applying de novo sequencing to identify circulating miRNAs associated with BC clinical outcome, lower levels of miR-375-3p significantly correlated with not achieving pCR in HER2-positive BC patients receiving doxorubicin/cyclophosphamide treatment followed by carboplatin and nab-paclitaxel plus trastuzumab [54].

Multiple studies are concordant on the possible association between circulating miR-125b-5p and NAC response. In fact, circulating baseline levels of miR-125b-5p were found to be higher in non-responders (defined as stable or progressive disease) compared to responders (defined as partial or complete response; *p* = 0.008) in serum samples of 56 BC patients with invasive ductal carcinoma undergoing four to six cycles of NAC with 5-fluorouracil, epirubucin, and cyclophosphamide [33]. Similarly, a Chinese study involving 118 patients diagnosed with stage II/III BC and undergoing four to six cycles of NAC with docetaxel, epirubicine, and cyclophosphamide showed that significantly higher levels of serum miR125b-5p were present in patients who had stable or progressive disease compared to those that had reached partial or complete response [30]. 

The identification of biomarkers that can be repeatedly tested through non-invasive approaches could provide the possibility of analyzing real-time information on disease evolution during treatment [55] (Table 3). In this context, many studies have aimed to identify the predictive biomarkers of NAC response by monitoring miRNA dynamic changes on multiple blood samples collected during therapy.

Again, one of the most investigated circulating miRNAs is miR-21-5p. A prospective clinical trial by Davey MG et al. evaluated the possible role of circulating miRNAs in decision making for NAC [56]. Blood miRNA levels were measured at diagnosis (Timepoint 1, or T1), and after two cycles of NAC (T2) in a total of 120 patients (59 luminal A, 21 luminal B, 15 HER2-positive, 25 TNBC). In the overall cohort, no circulating miRNAs were associated with response to NAC, but decreased or increased miR-21-5p levels trended to significance as associated with treatment response in specific BC subtypes. A second analysis evaluated levels of serum miRNAs during NAC in 83 HER2-positive early BC patients treated with four to six cycles of taxane-carboplatin plus trastuzumab [48]. Serum samples were collected before treatment, at the end of the second cycle, and at the end of therapy. The results showed that serum miR-21-5p levels in clinical responders were significantly lower at the end of the second cycle and at the end of therapy compared to baseline level (*p* < 0.001 for both), while there was no significant difference in non-responders. Dynamic circulating miR-21-5p levels were also investigated by Liu B. et al. in 118 patients affected by early HER2-negative BC [30] receiving four to six cycles of NAC with an association of docetaxel, epirubicin, and cyclophosphamide (TEC regimen). Blood samples were collected before treatment, at the end of the second cycle, and at the end of NAC. In line with the abovementioned results, the mean miR-21-5p level was lower during and after NAC than at baseline in responders (*p* = 0.016 for both), but not in non-responders. These results suggested that a decreased level of serum miR-21-5p detected after the second NAC cycle (compared to baseline) could be able to predict responder patients. 

Besides miR-21-5p, other circulating miRNAs have been investigated for their dynamic changes during NAC. The abovementioned prospective trial performed by Zhang Z. et al. evaluated circulating miRNAs in 37 luminal B early BC patients [40]. Blood samples were collected at baseline, and after two/four cycles of NAC. The circulating miR-210-3p levels during NAC were increased in non-responders, while the authors did not find a significant change in responders. In addition, significantly higher plasma miR-210-3p levels were observed in the non-pCR group than in the pCR group. Circulating levels of miR-210-3p associated with sensitivity to trastuzumab were evaluated in another trial involving 29 patients with HER2-positive early BC [29]. Patients received four cycles of taxanes followed by four cycles of anthracycline-based chemotherapy plus trastuzumab. Plasma samples were collected preoperatively and in the second postoperative week. The mean baseline level of plasma miR-210-3p was higher in samples from patients with residual disease than in the pCR group (*p* = 0.0359).

In the above-mentioned study by Liu B. et al. analyzing the dynamic predictive role of circulating miR-125b-5p [30], a significant association was found between miRNA level and NAC response; miR-125b-5p level was higher at all timepoints in non-responders than in responders; however, treatment did not induce statistically significant changes in miRNA levels in either group. Moreover, the analysis by Zhang Z. et al. reported that, in the luminal B/HER2-positive cohort, the levels of circulating miR-125b-5p remained relatively stable from the baseline through the first two/four cycles of NAC, in patients with both complete and partial response [40]. 

Recently, Todorova K. et al. performed RNA sequencing on plasma samples collected from 20 BC patients before and after the first cycle of NAC (combination of doxorubicin with cyclophosphamide). The authors showed an increased level of circulating miR-34a-5p after the first NAC dose in patients that did not achieve pCR [57]. Similarly, another analysis reported that the miR-34a-5p levels were significantly increased after two/four cycles of NAC (compared to baseline level) in luminal B responder patients, regardless of HER2 status [40]. A further study investigated serum miR-34a-5p levels during NAC in 86 HER2-negative BC patients [38]. All patients received six cycles of chemotherapy (anthracycline plus taxane-based regimen). Serum samples were collected at baseline, at the end of the second cycle, and at the end of NAC. The authors found that changes in miR-34a-5p levels during NAC were significantly associated with the chemotherapeutic responses. At the end of the second cycle and at the end of NAC, almost all responders had decreased serum miR-34a-5p levels compared to baseline (*p* < 0.001 for both). Finally, Zhu W. et al. evaluated the dynamics of circulating miR-34a-5p during NAC (i.e., epirubicin-paclitaxel regimen) [26]. In the HER2-positive and TNBC cohorts, plasma miR-34a-5p levels were significantly decreased in chemo-insensitive patients after the first two cycles of NAC (*p* = 0.027 and *p* = 0.006, respectively), while they remained stable throughout the course of treatment in chemo-sensitive patients (no statistically significant changes). 

In an analysis by Davey G.M. et al. [56], the authors found that increased miR-let-7a-5p levels (from baseline to after the second cycle of NAC) identified patients who achieved partial or complete response in the luminal HER2-positive cohort (*p* = 0.049), whereas in the luminal cohort reduced miR-let-7a-5p levels predicted achieving pCR (*p* = 0.037). Circulating miR-let-7a-5p was also analyzed in the NEOCENT trial, a phase III translational study in which 63 patients (ER-rich) were randomized 1:1 to receive chemotherapy (anthracycline-based regimen, followed by docetaxel in the case of poor response after the first three cycles) or endocrine therapy (letrozole) [58]. Blood samples were collected at baseline, after 8 weeks, shortly before surgery, and 6-monthly for 2 years; miRNA markers were assessed from baseline to the end of treatment for both arms. An increase in circulating miR-let-7a-5p level was associated with objective radiological response in both arms, but it was statistically significant only in the chemotherapy arm (*p* = 0.008).

**Table 2:** Main findings on circulating miRNAs as predictive markers in BC patients undergoing NAC.

miRNA	Sample	BC Patients	BC Subtype	Method	Predictive Findings	Ref
21-5p	Whole blood	114	All	qRT-PCR	Independent predictor of responseIn HR+, reduced levels in responders vs. non responders	[41]
	Plasma	72	HR+	q-PCR	Independent predictive factor of MP response to neoadjuvant chemotherapyReduced levels in responders vs. non-responders	[44]
	Serum	182	HR+, HER2+	qRT-PCR	Reduced levels correlated with sensitivity to NACIncreased levels correlated with resistance to NAC	[45]
145-5p	Whole blood	120	All	qRT-PCR	In HR- HER2+, lower levels predicted achievement of pCR (*p* = 0.027)	[56]
	Whole blood	114	All	qRT-PCR	In HR+, significantly lower levels in responders vs. non-responders (*p* = 0.033)	[41]
205-5p	Serum	182	HR+, HER2+	qRT-PCR	In HR+, increased levels predicted sensitivity to NACIn HER2+, low levels predicted resistance to NAC	[45]
	Serum	68	HR+	RT-PCR	Increased levels predicted resistance to NAC based on epirubicin plus paclitaxel (*p* = 0.003)	[53]
210-3p	Serum	37	Luminal B	qRT-PCR	Increased levels in pathological responders vs. non-responders (OR = 0.07, 95% CI = 0.01–0.45, *p* = 0.01)	[40]
	Plasma	29	HER2+	RT-PCR	Increased levels in residual disease vs. pCR (*p* = 0.0359)	[29]
222-3p	Plasma	109	All	qRT-PCR	In HR+ HER2-, upregulation predicted response to NAC (OR = 6.422, *p* = 0.049)	[26]
	Serum	65	HER2+	qRT-PCR	Reduced levels predictors of pCR (OR = 0.258, *p* = 0.043)	[59]
375-3p	Serum	37	Luminal B	qRT-PCR	In HER2-, lower levels predicted in pCR	[40]
	Serum	182	HR+, HER2+	qRT-PCR	In HR+, reduced levels predicted sensitivity to NAC	[45]
	Serum	42	All	qRT-PCR	In HER2+, high levels predicted resistance to NAC	[54]
19a-3p	Serum	68	HR+	RT-PCR	Increased levels predicted resistance to NAC-based on epirubicin plus paclitaxel	[53]
19b-3p	Serum	8	TNBC	RT-PCR	In TNBC, higher levels in no cCR to NAC	[27]
30b-5p	Plasma	20	HR+, TNBC	NGS	Upregulation predicted pCR	[57]
	Serum	8	TNBC	RT-PCR	In TNBC, higher levels in no cCR to NAC	[27]
34a-5p	Plasma	39	All	qRT-PCR	Higher levels in PD vs. SD/PR/CR (*p* = 0.03)	[37]
423-5p	Plasma	20	HR+, TNBC	NGS	Downregulation predicted pCR (*p* = 0.0005)	[57]
718	Serum	37	Luminal B	qRT-PCR	Lower levels in clinical responders vs. non responders (*p* = 0.031)	[40]
4516	Serum	37	Luminal B	qRT-PCR	Lower levels in clinical responders vs. non responders (*p* = 0.016)	[40]
146a-5p	Plasma	72	HR+	q-PCR	Independent predictive factor of MP response to neoadjuvant chemotherapyReduced levels predictors of response	[44]
26a-5p	Plasma	72	HR+	q-PCR	Independent predictive factor of MP response to neoadjuvant chemotherapyReduced levels predictors of response	[44]
127-3p	Plasma	20	HR+, TNBC	NGS	In TNBC, upregulation strongly predictor factor of pCR	[57]
195-5p	Whole blood	114	All	qRT-PCR	Reduced levels in responders vs. non responders	[41]
221-3p	Plasma	93	All	RT-PCR	Independent factor for chemoresistance	[50]
328-3p	Plasma	20	HR+, TNBC	NGS	Downregulation predicted pCR (*p* = 0.0019)	[57]
125b-5p	Serum	56	All	qRT-PCR	Higher levels in non-responders vs. responders (*p* = 0.008)	[33]

BC: breast cancer; cCR, clinical complete response; HER2: human epidermial growth factor 2; MP: Miller–Payne; HR: hormone receptor; NAC: neoadjuvant chemotherapy; NGS: next-generation sequence; OR: odd ratio; qRT-PCR: quantitative real-time polymerase chain reaction; pCR: pathological complete response; PD: progression disease; PR: partial response; SD: stable disease; Ref: reference.

**Table 3:** Statistically significant dynamic changes in circulating miRNA levels in BC patients undergoing NAC and association with response to treatment (*p* < 0.05). Up arrow indicates increased level from baseline. Down arrow indicates decreased level from baseline.

		Responders		Non-Responders		
miRNA	BC Subtype	After 1–4 Cycles of NAC	At the End of NAC	After 1–4 Cycles of NAC	At the End of NAC	Ref
21-5p	HER2+	↓	↓			[48]
	HER2−	↓	↓			[30]
145-5p	All	↓ in HER2+				[56]
210-3p	HER2+				↑	[29]
222-3p	Not specified			↑ in HR+/HER2−		[26]
34a-5p	HER2−	↓	↓			[38]
	All			↓ in HER2+ ↓ in TNBC	↓ in HER2+	[26]
let-7a-5p	All	↑ in HR+ HER2+ ↓ Luminal				[56]

Abbreviations: BC: breast cancer; HER2: human epidermal growth factor receptor 2; TNBC: triple-negative breast cancer; HR: hormone receptor; NAC: neoadjuvant chemotherapy; Ref: reference.

## 4. Prognostic Potential of Circulating miRNAs

MiRNAs have been extensively investigated for their promising role as prognostic biomarkers. However, despite several analyses suggesting them as potential prognostic tools in BC, the clinical application of these findings has yet to be verified [60]. A recent meta-analysis by Zhang et al. [61] analyzed 39 miRNAs with a prognostic value from 23 studies and found that 26 miRNAs were associated with survival outcomes. Although the study did not distinguish between tissue and circulating miRNAs, they identified miR-125b-5p, miR-21-5p, and miR-7-5p as the most frequently investigated ones with significant results. After reviewing the most recent literature, we identified seven circulating miRNAs significantly associated with prognosis in BC patients treated with NAC (Table 4).

**Table 4:** Main findings on circulating miRNAs as prognostic markers in BC patients undergoing NAC.

miRNA	Sample	BC Patients/ Healthy Controls	BC Subtype	Method	Prognostic Findings	Ref
21-5p	Serum	127/19	HER2+	qRT-PCR	Increased levels of circulating miR-21 before (*p* = 0.0091) and after (*p* = 0.037) NAC with trastuzumab and lapatinib showed a significant association with poor OS	[28]
	Serum	118/30	HER2−	qRT-PCR	Decreased expression from BL to FEN and from BL to SEN during NAC had better DFS (*p* < 0.001)	[30]
	Serum, blood	83/30	HER2+	qRT-PCR	Decreased serum expression from BL to the end of the second cycle and from BL to the end of NAC with trastuzumab correlated with better OS and DFS (*p* < 0.001)	[48]
	Serum	326/223	Not specified	qRT-PCR	High serum levels correlated with shorter RFS (*p* = 0.026) and DFS (*p* = 0.0033)	[62]
	Serum	75	Not specified	qRT-PCR	Increased expression (>nine-fold) was significantly associated with poor survival (*p* = 0.002)	[31]
34a-5p	Serum	86/20	HER2−	qRT-PCR	Decreased expression from the end of second cycle and the end of NAC to before NAC correlated with better DFS (*p* < 0.001)	[38]
	Blood	20	HR+, TNBC	NGS	Low level was prognostic for survival (*p* = 0.19)	[57]
125b-5p	Serum	118/30	HER2−	qRT-PCR	Lower expression at BL, FEN and SEN correlated with more favorable DFS (*p* < 0.001)	[30]
375-3p	Serum	182	HR+, HER2+	qRT-PCR	Low serum level (<0.15) correlated with lower 3-y RFS in luminal B patients	[45]
222-3p	Serum	65	HER2+	qRT-PCR	Low serum expression correlated with better DFS (*p* = 0.029) and OS (*p* = 0.0037)	[59]
4515p	Serum	27/36	Not specified	qRT-PCR	High levels at the time of diagnosis were associated with better DFS (*p* = 0.046)	[46]
182-5p	Serum	182	HR+, HER2+	qRT-PCR	High serum level (>5.5) correlated with lower 3-y RFS in luminal A patients	[45]

Abbreviations: BC: breast cancer; BL: baseline; DFS: disease-free survival; FEN: first evaluation during neoadjuvant chemotherapy; HER2: human epidermal growth factor 2; HR: hormone receptor; NAC: neoadjuvant chemotherapy; NGS: next-generation sequence; OS: overall survival; qRT-PCR: quantitative real-time polymerase chain reaction; RFS: recurrence free survival; SEN: second evaluation during neoadjuvant chemotherapy; Ref: reference.

As for diagnosis and prediction, among prognostic miRNAs, circulating miR-21-5p is one of the most extensively studied with the most robust data [28,30,31,48,62]. There is consistent evidence that low circulating miR-21-5p levels are associated with better outcomes, while higher levels are associated with worse outcomes. A study by Muller et al. was the first to investigate the effects of NAC with trastuzumab and lapatinib on serum levels of circulating miR-21-5p, miR-210-3p, and miR-373-3p in 129 HER2-positive BC patients compared with a cohort of 19 healthy controls. One of the aims of the study was to evaluate whether specific miRNA levels were associated with prognosis. Of the miRNAs investigated, only increased levels of circulating miR-21-5p before (*p* = 0.0091) and after (*p* = 0.037) NAC were associated with a statistically significantly worse overall survival (OS) [28]. In a similar cohort of HER2-positive BC patients treated with NAC combined with trastuzumab, Liu et al. evaluated the association of circulating miR-21 levels with survival. The authors analyzed blood and serum samples from 83 HER2-positive BC patients during different phases of NAC (at baseline, after two cycles, and at the end of treatment). They demonstrated that changes in serum miR-21-5p levels were significantly associated with survival outcomes. In particular, patients in whom circulating miR-21-5p levels decreased from baseline to the end of the second cycle and to the end of NAC showed better OS and disease-free survival (DFS) than patients with increased levels of this miRNA [48]. Similarly, an earlier study on HER2-negative BC confirmed that among the miRNAs investigated, a decrease in serum miR-21-5p and miR-125b-5p levels during NAC were associated with better DFS [30]. MiR-21-5p has also been proven to be associated with survival outcomes in two other studies conducted on BC patients (any subtype) [31,62]. In a study by Wang et al., conducted on more than 300 patients, high serum miR-21-5p levels were found to be an independent poor prognostic factor for both recurrence (HR 2.9; 95% CI 1.420–8.325; *p* = 0.008) and DFS (HR 2.7; CI 1.038–7.273; *p* = 0.003). In addition, patients with high miRNA levels had shorter recurrence-free survival (RFS) and disease relapse-free survival (DRFS) than patients with lower levels [62]. Accordingly, in another study, elevated miR-21-5p levels in blood samples collected before and after NAC from 75 BC patients was found to be significantly associated with poor survival (*p* = 0.002) [31].

It has been shown that circulating miR-34a-5p levels were associated with better survival outcomes in two studies conducted in HER2-negative BC. Liu et al. analyzed the serum miRNA levels of 86 patients during different phases of NAC and showed that changes in miR-34a-5p expression during treatment were significantly associated with treatment response and DFS. Furthermore, decreased miR-34a-5p levels between the end of the second cycle and the end of NAC compared to baseline levels were associated with improved DFS (*p* < 0.001) [38]. In a more recent study, blood samples from 20 patients collected before and after the first cycle of NAC were evaluated to investigate whether circulating exosomal miRNAs could predict pCR. The authors showed that decreased levels of circulating exosomal miR-34a-5p were associated with better OS [57].

Checkhun et al. analyzed the expression levels of circulating miRNAs in serum samples from 182 patients with luminal A and B BC undergoing NAC. The authors found that low serum miR-375-3p levels were associated with a lower rate of 3-year DRFS in luminal B patients. In contrast, higher levels of circulating miR-182-5p correlated with a lower 3-year DRFS rate in luminal A BC [45].

Regarding the circulating miR-200 family in BC, a study evaluating serum miR-222-3p levels in a cohort of 65 HER2-positive patients receiving anti-HER2 NAC showed that low serum miRNA levels were associated with better DFS (*p* = 0.029) and OS (*p* = 0.0037). Moreover, the study aimed to assess the association between circulating miRNA levels and trastuzumab-related adverse events. For the first time, serum miR-222-3p levels were found to be an independent protective factor for cardiotoxicity (*p* < 0.05) and anemia (*p* = 0.013), although the mechanism of action remains to be elucidated [59]. 

Finally, in a study conducted by Al-Khanbashi and colleagues, tissue and serum samples were collected from 27 BC patients undergoing NAC at four different timepoints (baseline, after the first and fourth cycle of doxorubicin/cyclophosphamide treatment, after the fourth cycle of docetaxel treatment) to assess the correlation between miRNA expression and different endpoints, including survival outcomes. The authors demonstrated that patients with high serum miR-451-5p levels at diagnosis were associated with better DFS (*p* = 0.046) [46].

## 5. Methodological Issues in Circulating miRNA Research

The presented results reflect the heterogeneity of available data on this subject. Indeed, these findings mainly come from single studies, involving different BC subtypes and/or patient characteristics (ethnicity, age, tumor stage, grade), different therapeutic regimens, and different definitions of responders and non-responders, thus limiting result comparison. In addition, accordingly to the literature, the majority of the discrepancies could be due to pre-analytical and analytical variables, as well as to patient-related factors that can generate artifacts, thus prejudicing the quantification of circulating miRNAs [20,63,64,65,66,67]. 

Specifically, one of the major critical issues is represented by the sample itself, including the type (plasma, serum, whole blood), collection (heparin, citrate, ethylenediaminetetraacetic acid, Paxgene tubes, sample handling), storage, processing (miRNA extraction method, timing of extraction), and blood cell contamination in sample preparation. In fact, it has been previously demonstrated that miRNAs contained in blood cells may have an influence on circulating miRNA analysis [68,69,70,71,72]; thus, different biological fluids show different circulating miRNA levels and whole blood may be strongly contaminated by blood cell miRNAs. In particular, the effect of haemolysis and the consequent release in the circulation of red cell miRNAs in impairing circulating miRNA analysis have been deeply investigated and proven [69,70,71,73]. In addition, blood cell miRNA contamination may also occur during sample collection, due to different centrifugation protocols used to separate plasma and serum from whole blood [74] and processing. For example, the correct choice of the anticoagulant for blood collection may, at least in part, prevent the lysis of red blood cells (e.g., citrate may trigger haemolysis) [75].

Another critical issue in circulating miRNA analysis that may, at least in part, explain the inconsistency among studies is the detection method used and the lack of a robust and standardized method for data normalization [64,65,66,67]. Since miRNA discovery, different quantification methods have been developed for miRNA analysis (e.g., quantitative real-time polymerase chain reaction (qRT-PCR), next-generation sequence (NGS), miRNA microarrays). Nowadays, qRT-PCR is the “gold standard” method since it allows a medium/high throughput and sensitive quantification of miRNAs. However, it can detect only annotated miRNAs, and the amplification step may affect miRNA quantification. NGS is a high-throughput method allowing the identification of both known and novel miRNAs with high sensitivity. However, it needs a larger quantity of each sample to be processed, it is the most expensive method, and it requires deep bioinformatics analyses. MiRNA microarrays are able to conduct high-throughput analysis on thousands of known and annotated miRNAs, but need to be constantly updated, thus impairing the comparison of results obtained with different platform versions. In addition, compared to NGS and qRT-PCR, miRNA microarrays show lower dynamic range and specificity [64,65,66,67,76].

Normalization strategy is a key issue for all platforms used for circulating miRNA quantification. Different endogenous miRNAs have been proposed as possible intrinsic controls for circulating miRNA analysis [19,72], and, among them, miR-16-5p is the most commonly used. However, it has to be considered that erythrocytes have high levels of miR-16-5p; thus, haemolysis may impair its use as an internal control [69,70,71,73]. Other normalization methods have been proposed (e.g., spiked-in synthetic RNAs, global normalization, quantile normalization); however, to date, a validated standardized normalization strategy has not yet been globally adopted. In addition, we have to consider that the normalization method may depend on the detection method used, thus further reducing the ability to compare results from different studies.

Finally, we have to highlight that in some of the analyzed studies it is not specified which strand of miRNA (-3p or -5p) is under investigation and, therefore, the authors refer to the miRBase alias used for the guide strand. However, considering that it has been demonstrated that both strands can be incorporated into the miRISC complex depending on tissue or cell type and act in gene regulatory networks [8], specific information about the strand is fundamental for adequate result comparison across different studies.

On the other hand, considering patient-related factors, as stated above, we should note that many results derived from single studies or from clinical studies enrolling a low number of patients, with no well-defined inclusion criteria [20,24,72], and with heterogeneous treatments. Moreover, we have to consider that circulating miRNA levels could be affected by various physiological conditions and/or comorbidities, such as obesity and diabetes, and these have to be taken into account during analysis [77,78]. In addition, different studies have shown that circulating miRNA levels may be affected by individual factors, such as age, race, drug assumption, smoking habits, diet, and physical activity [63,79,80,81,82,83,84,85]. Finally, we have to point out that BC is a heterogeneous disease, which may, at least in part, further explain the inconsistency of the results [86].

## 6. Conclusions

To date, besides few clinical genomic classifiers [87,88], there is no validated predictor associated with NAC benefit in terms of pCR and, ultimately, DRFS. Our review pointed out that circulating miR-21-5p and miR-34a-5p are the most promising non-invasive biomarkers for BC patients in the NAC setting and deserve further investigations. The collection of miRNAs from biological fluids at the beginning of NAC is simple and may aid in the identification of patients who will receive the greatest benefit. On the other hand, assessing miRNA levels during NAC is straightforward and faster than radiological assessment and may serve as a red warning in cases of poor response, particularly when it is clinically challenging to assess. However, due to the disagreement between studies, further clinical trials with more precise patient inclusion criteria and more standardized methodological approaches are definitely needed to better define the predictive/prognostic ability of these promising non-invasive biomarkers in anticipating the treatment response and outcome of BC patients undergoing NAC.
